# Association Between Coffee Consumption and Academic Performance Among Second-Year Medical Students

**DOI:** 10.7759/cureus.103534

**Published:** 2026-02-13

**Authors:** Smruti S Petkar, Sangeeta Dabhade

**Affiliations:** 1 Department of Pharmacology, B. J. Government Medical College (BJGMC) Pune, Pune, IND

**Keywords:** academic impact, academic performance, coffee consumption, coffee intake, medical education, medical students, sleep pattern, study duration, study habits

## Abstract

Background and aim

Caffeine is one of the most widely consumed psychoactive substances, with coffee serving as a primary source among university students globally. Many medical students use coffee to counter fatigue, stay alert, and maintain focus during extended study sessions and examinations. Caffeine works mainly by blocking adenosine receptors, which promotes wakefulness and temporarily improves attention and responsiveness. However, excessive consumption can lead to anxiety, disrupted sleep, and reduced cognitive function, potentially affecting academic performance. Despite the popularity of coffee, its overall impact on academic outcomes among medical students remains uncertain. This study aimed to explore the relationship between coffee consumption and academic performance among second-year medical students following their prelims examination.

Methods

This cross-sectional observational study was conducted among second-year medical students to assess the association between coffee consumption and academic performance. A structured, self-administered questionnaire was used to collect data on coffee intake patterns, demographic variables, lifestyle factors, and self-reported academic indicators. Academic performance data were obtained from institutional records of preliminary examinations. Statistical analysis was performed using appropriate descriptive and inferential methods to evaluate associations between coffee consumption and academic outcomes.

Results

A total of 249 students were included in the analysis. Of these, 172 students (69.1%) reported regular coffee consumption, while 77 students (30.9%) were nonconsumers. The mean theory prelim examination score was higher among coffee consumers compared to nonconsumers. Students consuming one to two cups of coffee per day (96 students, 38.6%) demonstrated higher mean examination scores than both nonconsumers and those consuming three or more cups per day (76 students, 30.5%). Examination scores also differed significantly across categories of sleep duration and reported caffeine-related adverse effects.

Conclusions

Coffee consumption was associated with academic performance among second-year medical students. Moderate intake was linked to improved academic outcomes, while excessive consumption, particularly when accompanied by sleep disturbances, was associated with poorer performance. These findings highlight the importance of moderation, adequate sleep, and healthy study habits in optimizing academic performance.

## Introduction

Caffeine is a popular psychoactive substance with well-characterized neurostimulatory effects. It primarily acts on the central nervous system by antagonizing adenosine A1 and A2A receptors, thereby reducing inhibitory neurotransmission and increasing neuronal excitation. This mechanism leads to increased release of dopamine, norepinephrine, and acetylcholine, which enhance alertness and cognitive arousal [1]. Recent evidence suggests that caffeine can improve various aspects of cognitive functioning, including alertness, sustained attention, reaction time, and short-term memory, particularly during periods of fatigue or sleep deprivation. Moderate caffeine consumption has been shown to enhance vigilance and accuracy in cognitively demanding tasks, whether in academic testing or other circumstances [2]. However, these effects are dose-dependent, and excessive intake can result in cognitive instability, anxiety, and impaired executive functioning [3].

Despite widespread consumption, the relationship between coffee intake and academic performance remains inconsistent in the literature. Some studies report improved cognitive or academic outcomes with moderate caffeine use, whereas others demonstrate no benefit or even negative associations when consumption is excessive or accompanied by sleep disruption [4,5]. Although caffeine consumption among students has been widely studied, most investigations rely primarily on subjective outcomes, such as perceived alertness, sleep quality, or self-reported academic performance. Objective academic indicators derived from institutional examination records have been comparatively less studied. The present study, therefore, evaluates academic performance using official prelim examination scores to provide a more objective assessment of the association between coffee consumption and academic outcomes among medical students.

The primary aim of this study is to examine the association between coffee consumption and academic performance among second-year medical students following their prelims examination. The secondary objectives are to compare mean academic examination scores between coffee consumers and nonconsumers, to evaluate the association between different patterns of coffee consumption (frequency and quantity) and academic performance, and to identify any adverse effects associated with coffee consumption.

## Materials and methods

Study design

This cross-sectional observational study was conducted among second-year medical students enrolled during the academic year 2024 at a government medical college in Western Maharashtra. The study was carried out over one month following the theory prelims examination in January 2025. It was designed to assess associations rather than causal relationships.

Study population

All second-year medical students who appeared for the theory prelim examination during the study period were eligible. A total of 265 students were invited to participate via an electronic questionnaire as part of a universal sampling (Appendix A). Of these, 249 students responded, and after verifying the completeness of responses, all 249 participants were included in the final analysis. No participants were excluded after enrollment.

Ethical considerations

The study protocol was reviewed and approved by the Institutional Ethics Committee (approval ND-1024326-326). All participants provided electronic informed consent. Participation was voluntary, and confidentiality was maintained by anonymizing data prior to analysis.

Inclusion and exclusion criteria

Included were second-year medical students who had completed their prelims examination, were willing to participate, and provided complete questionnaire responses. Students with regular intake of other caffeinated stimulants (e.g., energy drinks) or medications affecting sleep or cognition (e.g., antidepressants, beta blockers, and benzodiazepines) were excluded from the study.

Data collection tool

A structured, validated, self-administered questionnaire was distributed electronically. The questionnaire was developed specifically for this study based on relevant literature and study objectives. Face and content validity were ensured through expert review by faculty members from the pharmacology department; formal psychometric validation was not performed.

The questionnaire included items assessing coffee consumption status, average daily intake (number of cups per day), and reasons for consumption. Additional items evaluated included average daily study duration, average nightly sleep duration, perceived cognitive effects of coffee intake, and self-reported caffeine-related adverse symptoms, including gastrointestinal discomfort, palpitations, anxiety, headaches, sleep disturbances, and other relevant effects. Coffee consumption was assessed in terms of frequency and quantity, defined as the number of cups consumed per day according to usual consumption patterns. Standardization of cup size or caffeine content was not undertaken. Sleep duration was self-reported as average hours of sleep per night. Study habits (hours/day) and caffeine-related adverse effects were self-reported through a pre-listed checklist. Academic performance was obtained objectively using prelim examination scores from institutional records. The theory examination was scored out of a maximum of 200 marks.

Statistical analysis

Data were entered into Microsoft Excel (Microsoft Corporation, Redmond, WA, USA) and analyzed using appropriate statistical methods. Continuous variables were summarized as mean ± standard deviation, and categorical variables were expressed as frequencies and percentages. Comparisons of mean theory prelim examination scores between coffee consumers and nonconsumers were performed using an independent samples t-test. Differences in academic performance across categories of coffee consumption frequency, sleep duration, and reported adverse effects were assessed using one-way ANOVA. A p-value of less than 0.05 was considered statistically significant.

## Results

Participant characteristics

Table 1 presents the baseline demographic characteristics of the respondents (total n = 249). The study sample was relatively homogeneous in terms of age (20.8 ± 1.1 years) and academic background, as all participants were from the same academic year and institution. The sex distribution included 134 male and 115 female students, reflecting a balanced population.

**Table 1:** Demographic and baseline characteristics of study participants (n = 249)

Variable	Value
Age (years), mean ± SD	20.8 ± 1.1
Male, n (%)	134 (53.8)
Female, n (%)	115 (46.2)
Coffee consumers, n (%)	172 (69.1)
Non-coffee consumers, n (%)	77 (30.9)
Average study duration (hours/day)	4.1 ± 1.6
Average sleep duration (hours/day)	6.2 ± 1.3
Theory prelim score (out of 200)	128.4 ± 22.7

Among the participants, 172 students (69.1%) reported regular coffee consumption, while 77 students (30.9%) reported no coffee consumption. The mean daily study duration was 4.1 ± 1.6 hours, and the mean nightly sleep duration was 6.2 ± 1.3 hours. The overall mean theory prelim examination score was 128.4 ± 22.7 marks (out of 200).

Demographic profiling was performed because certain variables, such as age and sex, can influence caffeine metabolism, stress response, and academic performance [6,7].

Coffee consumption and academic performance

The analysis of coffee consumers and nonconsumers, as shown in Table 2, indicated a difference in mean theory prelim examination scores. Coffee consumers scored higher than nonconsumers (131.6 ± 21.8 vs. 122.9 ± 23.4), and this difference was statistically significant (p = 0.041).

**Table 2:** Comparison of theory prelim scores between coffee consumers and nonconsumers Values are expressed as mean ± SD. An independent samples t-test was used. A p-value < 0.05 was considered statistically significant. Statistically significant p-values are indicated by an asterisk (^*^).

Coffee consumption status	n	Mean academic scores ± SD	Test statistic	p-Value
Coffee consumers	172	131.6 ± 21.8	t = 2.06	0.041^*^
Nonconsumers	77	122.9 ± 23.4	-	-

Further subdivision based on the frequency of coffee consumption (Table 3) revealed that students with moderate intake (1-2 cups/day) tended to achieve higher academic scores (124.1 ± 22.9) compared to nonconsumers (122.9 ± 23.4) and those who consumed excessive amounts of coffee. Differences in examination scores across these categories were statistically significant (ANOVA, p = 0.018). 

**Table 3:** Theory prelim scores according to frequency of coffee consumption Values are expressed as mean ± SD. One-way ANOVA was used. A p-value < 0.05 was considered statistically significant. Statistically significant p-values are indicated by an asterisk (^*^).

Coffee intake category	n	Mean academic scores ± SD	Test statistic	p-Value
Nonconsumers	77	122.9 ± 23.4	-	-
Moderate consumers (1-2 cups/day)	96	136.8 ± 19.6	F = 4.29	0.018^*^
Heavy consumers (≥3 cups/day)	76	124.1 ± 22.9	-	-

Effect of sleep duration on academic performance

Sleep duration emerged as an important modifying factor in the relationship between coffee consumption and academic performance. Students who reported sufficient sleep (≥7 hours per night) in combination with coffee consumption achieved higher examination scores compared to those who were sleep deprived (<5 hours). These values are presented in Table 4. Differences across sleep duration categories were statistically significant (ANOVA, p = 0.009).

**Table 4:** Effect of sleep duration on academic performance among coffee consumers Values are expressed as mean ± SD. One-way ANOVA was used. A p-value < 0.05 was considered statistically significant. Statistically significant p-values are indicated by an asterisk (^*^).

Sleep duration	n	Mean academic scores ± SD	Test statistic	p-Value
≥7 hours/night	58	139.2 ± 18.7	-	-
5-6 hours/night	79	130.5 ± 20.4	F = 4.83	0.009^*^
<5 hours/night	35	118.6 ± 21.9	-	-

Adverse effects

Reported negative effects of coffee consumption included gastrointestinal discomfort, palpitations, nervousness, headaches, and sleep disturbances. Students who experienced minimal or no adverse effects generally maintained steady academic performance, whereas those reporting significant sleep disturbances or anxiety-related symptoms had relatively lower examination scores (Table 5).

**Table 5:** Adverse effects of coffee consumption and academic performance Values are expressed as mean ± SD. One-way ANOVA was used. A p-value < 0.05 was considered statistically significant. Statistically significant p-values are indicated by an asterisk (^*^).

Adverse effects reported	n	Mean academic scores ± SD	Test statistic	p-Value
No adverse effects	103	136.9 ± 19.4	-	-
Mild adverse effects	46	128.7 ± 20.6	F = 3.98	0.021^*^
Significant sleep/anxiety symptoms	23	117.3 ± 22.1	-	-

Coffee consumption status, frequency of intake, sleep duration, and reported adverse effects were all associated with differences in theory prelim examination scores. Statistically significant differences were observed across multiple comparisons, as detailed in Table 2, Table 3, Table 4, and Table 5. Given the cross-sectional design, these findings represent associations rather than causal relationships and should be interpreted cautiously, considering the potential for residual confounding.

## Discussion

The current study examined the relationship between coffee consumption and academic performance among second-year medical students and found that coffee use was associated with differences in examination results. Students who reported moderate coffee consumption performed better academically compared to nonconsumers and heavy consumers. This finding aligns with other recent studies in university populations, which also suggest potential cognitive benefits of controlled caffeine use during periods of academic stress [4,6]. However, many earlier studies relied on self-reported academic outcomes or subjective cognitive measures, whereas the present study used institutional examination scores as objective indicators, potentially strengthening the reliability of the observed associations.

The findings indicate that moderate coffee consumers achieved higher academic performance than both nonconsumers and excessive consumers. Sleep duration and consistent study habits were associated with differences in academic performance across coffee consumption categories. Students experiencing adverse effects, such as anxiety or sleep disturbances, demonstrated poorer academic outcomes.

Short-term benefits of caffeine may be offset by physiological or psychological adverse effects. Recent observational studies in student populations have reported similar associations between caffeine-related adverse effects and reduced academic or cognitive performance [8].

The observed association was not consistent across all consumption patterns, suggesting that coffee intake alone does not determine academic success. These findings are consistent with existing evidence indicating that the effects of caffeine on academics depend on behavioral and physiological factors, including sleep quality, stress levels, and study habits [3]. The variability of results across different consumption levels contributes to inconsistencies reported in the literature.

This study demonstrated that moderate caffeine intake enhances alertness and attention, whereas excessive consumption can impair performance due to anxiety and sleep disruption, consistent with findings by Cappelletti et al. (2015) [3] and Zunhammer et al. (2014) [9].

Poor academic performance is linked to sleep deprivation and inconsistent study patterns, regardless of caffeine consumption. These observations align with current literature emphasizing the importance of sleep quality and regular study habits in learning efficiency and examination performance, which may confound the relationship between caffeine use and academic outcomes [10,11]. These factors may partly explain the superior performance of students with moderate caffeine consumption, particularly under high academic stress [12]. Acute caffeine use may temporarily reduce fatigue and improve task efficiency, which can be beneficial during exams.

Although caffeine may confer short-term benefits, long-term or excessive consumption has been associated with negative cognitive and psychological effects, including anxiety, restlessness, poor concentration, and dependence. Recent studies highlight a dose-related effect: moderate doses improve cognitive function, while higher doses impair executive control and working memory [8,9]. These results suggest that the positive effects of coffee depend on maintaining an optimal level of consumption. Additionally, previous studies have shown that caffeine intake can alter sleep quality and daytime functioning in student populations [13-15].

Sleep duration appears to be a key factor in the relationship between coffee consumption and academic performance. Adequate sleep is essential for memory consolidation, learning efficiency, and cognitive functioning. Even among coffee consumers, students with sufficient sleep showed better academic outcomes [10].

Conversely, abnormal caffeine consumption, particularly excessive or late-day intake, can disrupt sleep by delaying onset and reducing total duration. Sleep deprivation adversely affects attention, working memory, and information retention, ultimately impairing examination performance. Recent research indicates that the cognitive benefits of caffeine in the short term may be outweighed by the negative effects of inadequate sleep, particularly when long-term sleep disruption occurs [11,12].

Limitations

The study, however, has some limitations. The cross-sectional design does not allow the establishment of causal relationships between coffee consumption and academic performance. Certain potential confounding factors, such as alcohol intake, smoking status, stress levels, and baseline academic aptitude, were not specifically assessed in this study. These variables may independently influence academic performance and could partially account for the observed associations. Future studies incorporating a broader range of lifestyle, psychological, and academic baseline parameters would help provide a more comprehensive understanding of the determinants of academic performance among medical students. Additionally, although the face and content validity of the questionnaire were ensured through expert review, formal psychometric validation was not performed, which may limit the generalizability of the self-reported responses. Moreover, coffee intake and other associated variables were self-reported and thus may be subject to bias, and variations in serving size, preparation method, and caffeine concentration may have influenced the estimation of caffeine exposure. Although institutional examination scores were used as objective academic indicators, detailed information regarding individual examiner allocation and variability was not specifically assessed. As a single-center study conducted in one medical college, the generalizability of the findings to other student populations may be limited. Despite these constraints, the findings provide useful information on caffeine consumption habits and their possible role in education and serve as a basis for future longitudinal and interventional research with standardized caffeine exposure assessment and broader evaluation of lifestyle, psychological, and academic baseline factors. Such studies may offer a more comprehensive understanding of how caffeine interacts with sleep, stress, and study behaviors in academic settings.

## Conclusions

Coffee consumption among second-year medical students shows a dose-related association with academic performance. Moderate intake may enhance alertness and support performance, whereas excessive consumption, particularly when accompanied by sleep disturbances, can negatively affect academic outcomes. Coffee should be considered a supportive tool within a broader framework of healthy sleep and study habits. However, causal relationships cannot be inferred due to the cross-sectional design. These findings highlight the importance of balanced caffeine use alongside adequate sleep and effective study practices. Awareness programs on safe caffeine consumption and sleep hygiene should be incorporated into medical education. Future longitudinal and interventional studies are recommended to establish causality.

## Appendices

Appendix A: Questionnaire

I consent to using my provided information for the same: ____________

Signature of participant

1. Demographic information

Name:

Age:

Gender:

Roll number:

2. Coffee consumption

Do you consume coffee? (Yes/No)

How often do you drink coffee? (Occasional, Regular, Daily)

On average, how many cups of coffee do you drink per day? (Mention number of cups) 1, 2,3, 4, 5, more than 5

At what time of the day do you usually drink coffee? (Morning/Noon/Evening/Night)

What is your purpose for consuming coffee?

Alertness/concentration/taste, others _____________

3. Study habits

How many hours do you study per day on average?

Do you study at night? (Yes/No)

4. Sleep patterns

How many hours of sleep do you get per night on average?

Do you feel well-rested when you wake up in the morning? (Yes/No)

Do you have insomnia?

If yes, how many hours after consumption of coffee does it increase alertness? __________________________________________

Do you experience daytime sleepiness? No/Sometimes/Occasionally/Regularly

5. Adverse effects

Do you experience any of the following side effects after consumption of coffee?

1. Nausea/Vomiting/Headaches

2. Palpitations/Restlessness/Jitters

3. Diarrhea/Gut disturbances/Acidity

4. Frequent urination

5. Others ___________________

Have you considered decreasing/stopping coffee intake because of the side effects? Yes/No

Do you consume any other caffeinated beverages apart from coffee?

Mention name: ___________________________________

Frequency of consumption

6. Academic performance

Do you feel that consumption of coffee improves your alertness/wakefulness during examinations?

Do you think consumption of coffee helps you concentrate while studying? Yes/No

Do you feel that consumption of coffee will help you improve your overall academic performance?

Marks obtained in the recent prelim examination:
